# Therapœia

**Published:** 1826-01

**Authors:** 


### THERAPŒIA.

Pharmaca nulla valent, nisi quæ sint commoda causis.

1. *Hepatic Ileus—or, Dry Belly-ache.** Dr. Musgrave has carried all the energy and zeal of his native climate into the enervating atmosphere of tropical regions. It is to such men we are to look for increase of our knowledge, not only as respects West Indian diseases, but many of those which, under a milder sky, assume less terrible and more equivocal forms. That peculiar affection which has long been known in the Antilles by the name of "dry belly-ache," appears to be one of very frequent occurrence, at least of late years, in Antigua. Attentive observation of its phenomena has led to a conviction in our author's mind, "that the published description of its symptoms is inaccurate and defective—its real nature imperfectly understood—and its treatment very generally conducted on erroneous principles." The following description we shall give in Dr. Musgrave's own words, as they cannot be abridged without detriment to the original.

"The patient, if advice be called for at an early stage of the attack, complains only of an uneasy sense of distention behind the ensiform cartilage, of loss of appetite, a feeling of listlessness, and disturbed rest. His general appearance betrays little of indisposition beyond a slight expression of anxiety in the countenance; his tongue is clean; his skin cool and comfortable; his pulse unaltered, or, if altered at all, it is rather reduced in frequency and augmented in strength; his urine, if examined, is probably found high-coloured and scanty; but, what is especially remarkable, he affirms his bowels to be *regularly and sufficiently open*, and his evacuations of natural appearance. If his memory be particularly directed to the state of his health for a few preceding days or weeks, he will admit, (at least, it has happened so to me in nine cases out of ten,) that without being actually unwell, his ordinary mental elasticity and bodily vigour have, during that period, been more or less impaired, and that he has been conscious of a degree of uneasiness in some part of the right hypochondrium, so trifling, however, as to be considered of no importance. This state of things may continue with little variation for a week or more, and, if proper measures be taken, the complaint may be removed before it makes farther progress. But if these premonitory symptoms be disregarded, as is too often the case when the individual is engaged in business, or sometimes, in spite of all that we can do, if they have existed for two or three days before

* Dr. Ant. Musgrave, of Antigua. Med. Repos. Nov. 1825.

our advice is called for, the uneasiness I have described gradually amounts to pain which, becoming more and more acute, shoots, in a majority of examples, upwards across the chest, still farther misleading the patient or inexperienced practitioner as to the real nature of the approaching illness. About this time, nausea will first occur, followed by vomiting of porraceous matter, and subsequent annoyance from twisting pain about the umbilicus; but even now medicines are occasionally retained, and a smart purgative will often act with tolerable effect in discharging the ordinary contents of the canal. Very shortly, however—generally within twenty-four hours after the accession of severe abdominal symptoms—the constipation commences with distressing irritability of stomach; but I think I have remarked, that after the obstruction becomes complete, the matters thrown off in retching are no longer bilious, but consist entirely of the liquids which have been taken into the stomach mixed with its own secretions and those of the fauces: indeed, I have been accustomed to regard the supervention of *bilious* vomiting in the more advanced stages as a peculiarly favourable indication.

" These aggravations are accompanied by a slower, harder, and more expanded pulse. I have known it less than fifty, with occasional intermissions, the artery feeling under the finger extremely large and firm. The tongue is still clean—indeed, it continues tolerably so throughout the whole course of the attack, the most common morbid appearance being that of frothy vesicles over a whitish surface, which can scarcely be said to be coated. The patient complains of thirst, is exceedingly restless in whatever position he may be placed, and his countenance betrays the most marked anxiety and alarm. The abdomen, in successful cases, rarely becomes tumid to any considerable extent. Pressure is borne without material inconvenience; and the only peculiarity which minute examination detects, is a degree of hardness from rigidity of its muscular parietes. The knotty lumps and spasmodic retraction of the navel towards the spine, I confess myself to have seldom seen; and I strongly suspect that this part of the description of dry belly-ache, as a tropical disease, has been rather copied by one author from another, than derived from clinical observation, it being applicable chiefly to the colica pictonum of Great Britain. The abdominal pain, however, is often highly acute, and the general distress of the patient indescribably great, equalling any thing I have ever witnessed in diseases of fatal termination; but, in my opinion, this distress is not entirely referrible to *pain*, since, being clearly spasmodic in its nature, this last occasionally subsides, affording intervals of comparative ease. Yet the inquietude is unrelieved, and sleep, even for one hour, is vainly courted by the unfortunate sufferer. The cause of this incessant restlessness, whether pain be present or not, is to a superficial inquirer obscure and perplexing; and it will, therefore, be part of my object to attempt an explanation of it when I treat of the theory of the symptoms.

" Hepatic ileus, or that species of colic which I have undertaken to delineate, becomes now completely developed. The bowels obstinately resist the most powerful purgatives. Hiccough is often superadded to the other annoyances. Injections are with difficulty retained, and returned untinged by feculent or other admixture, and the stomach rejects almost every thing. Epileptic fits are also no uncommon occurrence at this stage, when the patient is of broken constitution, the consequence of repeated attacks.

"This period of dreadful suffering may be protracted for several days, each adding, of course, to the degree of danger to be apprehended; and the judicious practitioner will be careful to mark the most trivial change, as he must be aware that every thing depends upon his prompt discrimination.

"While the pulse remains below one hundred in number—the skin continues cool—the tongue retains its moisture—the abdomen is felt free from tympanitic distention, and moderate pressure is borne without shrinking,—we have every right to hope that matters may yet terminate favourably, whatever may be the severity or duration of the other symptoms. But contraction and acceleration of the pulse, with febrile heat and a parched tongue, the constipation remaining unsubdued, are the heralds of an impending assault, which, if not vigorously resisted, menaces the vital powers with speedy destruction. The abdomen soon enlarges, and can be examined only by giving pain. The patient is constantly tossing himself from one part of his bed to another; he earnestly implores relief from intolerable sufferings, which appear to him to be the harbingers of death; his thirst is insatiable, although every thing he swallows is rejected;* hiccough harasses him without intermission; his pulse becomes smaller and more rapid; cold perspirations bedew the extremities; respiration is hurried; the pain is gradually felt less and less acutely, till it ceases altogether. Enormous liquid motions, highly offensive, are squirted from the anus with considerable force, and life is extinguished within a very few hours from their commencement.

"Such is the usual progress to death from dry belly-ache; but let us turn to the pleasing reverse, and trace the several steps to recovery in more fortunate examples.

"The first decidedly favourable indications are, an abatement of the irritability of stomach and less frequent jactitation, which in my own practice have been generally simultaneous with mercurial fœtor of the breath and swelling of the gums. This amendment generally takes place on the third or fourth day from the commencement of total obstruction; and, about the same time, we find the seat of the pain described as having almost imperceptibly descended to the hypogastric region (the epigastrium and both hypochondria being comparatively at ease): generally, before many hours have subsequently elapsed, the

"* *Stercoraceous* vomiting is rare, as it can only occur in neglected cases; but I have at this stage repeatedly observed dark matters, mixed with the fluids thrown up, resembling the depraved secretions which are afterwards passed by stool."

injections, which have hitherto been returned exactly as thrown up, are observed to bring with them some colouring matter which they have dissolved while in the gut. Another will present small portions of a dark-green or darker pitch-like substance floating on its surface. A desire for spontaneous evacuation will shortly afterwards produce a scanty motion composed of several portions of the same kind; and if the advantage gained be assiduously improved, a quantity of this bilious colluvies will be discharged for several days, which must be inconceivable to those who have never seen or treated the disease, the progress to health keeping pace with the gradual improvement in the nature of the evacuations, till nothing but debility and a degree of ptyalism remain. It may be as well to remark, that as the alvine discharges become, in succession, less inspissated, and assume more decidedly the appearance of vitiated bile, they are mixed with a thicker portion (obviously also a depraved secretion from the liver), which has not inaptly been compared to the fat of our West Indian crab; but scybalous masses are never passed upon the first removal of the obstruction, except in cases where the early stage has been entirely neglected; because, as I have already said, we find no difficulty, when applied to in time, in emptying the bowels of their natural contents, even though we fail to arrest the progress of the disease. The inference, therefore, is undeniable, that intestinal or fæcal accumulation can never be admitted among the exciting causes of this species of the complaint." 444.

Attentive observation and reflexion during the last five or six years have convinced our author, that the "dry belly-ache" is not an idiopathic affection of the intestines, "but one which originates exclusively in the liver"—consequently, that it differs, in some essential particulars, from the colica pictonum of the mother country. So far back as the year 1817, Dr. M. became convinced that low, damp situations did, at certain seasons of the year, and under particular circumstances, produce this derangement of the hepatic functions—but he is also convinced, that the most fertile source of the complaint is the prevailing consumption of rum, "which undoubtedly acts as a specific poison on the liver." Our author has, from personal observation, verified the fact, that wine-drinkers in the West Indies escape visceral diseases in an infinitely greater proportion than those who addict themselves to grog.

Dr. M. is not prepared to point out, with minuteness, the characters which distinguish hepatic ileus from the colica pictonum; the latter disease, as produced by lead, being comparatively rare in the West Indies, and, when it does exist, being generally complicated with the other, in consequence of the addiction of painters to the use of rum in excess. Still he thinks it will be discovered by the attentive observer that—"constipation from the very outset of the complaint—a protracted duration—a greater degree of spasmodic action, as well of the intestines as of the abdominal muscles, and the appearance of the stools when procured, furnish, during our earlier attendance, marks sufficiently distinctive of painter's colic, as contrasted with hepatic ileus." As paralysis has never supervened upon the latter disease, in his experience, this may also tend towards the discrimination. The following is our author's reasoning on the ratio symptomatum of the hepatic ileus.

"But to resume my subject. Admitting that I am correct in attributing the particular species of ileus in question to such a derangement in the hepatic functions as produces an increased and depraved secretion of viscid bile, the *ratio symptomatum* appears to me to be abundantly simple, and to afford a ready explanation of circumstances which, upon any other supposition, are contradictory, and not to be reconciled. The dull sense of weight about the right hypochondrium, which, as was formerly stated, is repeatedly found to precede the attack for days or weeks, marks incipient interruption of the regular and healthy process of biliary secretion. The fluid received by the pori biliarii becomes gradually increased in quantity, vitiated in quality, and of a thicker consistence. Hence its course through the ducts must, to a certain extent, be impeded, and time be given for absorption to effect still greater inspissation, which, of necessity, causes it to flow more and more slowly till it can no longer obtain a passage, and the obstruction becoming complete, the liver is left in a state of dangerous infarction.

"This view of the subject being adopted, the real seat of the pain, described as being behind the ensiform cartilage, is at once discovered. It evidently is to be referred to the ductus communis, cystic duct, and gall-bladder, distended with an inspissated mass; while the harassing sense of inquietude and constant jactitation may be explained as consequent upon the hepatic duct and pori biliarii being gorged with the offensive matter, which is still secreted, although an outlet no longer exists.

"Again, assuming these principles to be tenable, the fact that the bowels are easily moved, in the first instance, is explained with equal facility. The disease at this stage is *not in the intestines*. It has not yet proceeded beyond *the liver and its appendages*, and purgatives will therefore produce their usual effect. Subsequently, however, the irritation is communicated from the ductus communis to the duodenum. Doubtless, also, small quantities of this acrid secretion may occasionally escape into the latter, which, throwing the intestines into a state of spasm, produce abdominal pain, irritability of stomach, obstinate constipation—in a word, all the usual phenomena of ileus; and the quantity of this colluvies which is subsequently voided must be regarded not only as the previous accumulation in the excretory ducts, but as the continued product of an enormous gland stimulated into increased action as well by the original causes as, to a certain extent also, by the effect of remedies employed for the cure of the disease." 448.

We cannot follow our author through all his arguments in support of the hepatic origin of the disease; but have no hesitation in saying, that they are ingenious, if not conclusive.

Although a tropical climate presents many obstacles to pathological investigations, yet our author has laudably availed himself of every opportunity to test the truth of his theory by post mortem researches. "Hepatic congestion and infarction have been uniformly detected in *all*, and marks of intestinal inflammation and its consequences in a *majority* of examples, since, whatever may be its source, the termination of the disease is very commonly by mortification of some part of the canal."

"The etiological view of the symptoms which I have ventured to submit presents, at once, three principal indications:—to remove the obstruction, if possible, by such medicines as may, in the first instance, stimulate the ducts through the medium of the intestine; to excite the liver to a more healthy action; and, at the same time, to obviate any tendency to inflammation which may chance to occur during the progress of our remedial measures. Calomel, both purgative and cholagogue, must be eminently calculated to answer two of these indications; and hence, the practitioner who most relies upon its *well-regulated* administration will, in the main, have his confidence amply repaid. Perhaps I may even go so far as to say, that this disease, of all others, supplies the most reasonable excuse for a degree of freedom in the employment of mercury, which must appear the extreme of temerity to those unacquainted with what we have to encounter, for the question is too often one not of option but of painful necessity, and resolves itself into the choice of supinely witnessing the otherwise certain approach of death, or of boldly fronting the odium to be incurred by a rapid production of ptyalism; the fact being indisputable, that in the severer cases relief is seldom, if ever, obtained till the gums become swollen, but that, as soon as they do become so, the obstruction, whatever may be its source or situation, can be readily overcome. I have said, the *odium* to be incurred by the latter alternative, because it is undoubtedly true that the reputation of the physician is far less liable to detraction in the event of the death of his patient, than by his being judiciously rescued from danger, provided it so happen that the babbling effects of this powerful agent divulge, during recovery, that it has contributed to the accomplishment of this object. It therefore requires no ordinary share of moral courage to enable a man to keep the narrow path of his duty, in the face of such evident danger to his interest.

"It is certainly too much the fashion among physicians in the British metropolis to decry the use of mercury, and to insinuate to persons about to sail for this part of the world, and already sufficiently alarmed, that they will place themselves in greater jeopardy by following the prescriptions of those whom they must of necessity consult, than by the effects of the climate itself; and I give my transatlantic brethren credit for purity of intention, and for being totally unconscious of the serious mischiefs they occasion by circulating such an opinion; but it does not, on that account, become less imperatively my duty to expose such mischiefs, and, by doing so, to make them fully aware that prejudices are thus incautiously instilled into the minds of our visitors, which ultimately lead to their premature death, and not improbably the consequent ruin of a numerous and unprotected family. This is no suppositious or barely possible case. I write with a melancholy example conspicuously in view, which occurred not many days since, in the person of a respectable and highly valued mercantile member of this community, who had been about twelve months in the island. On Saturday evening febrile symptoms betrayed themselves; but Sunday, Monday, and Tuesday, were allowed to glide away without application for medical advice, from a dread of calomel so deeply rooted as not to be overcome. On Wednesday, he was visited, but medicine at this period proved unavailing; and on the Saturday night following, he left a wife and several children to deplore his loss, when they were fondly expecting his immediate return to an establishment in England.

"But the illiberality of these insinuations is the more provoking and unpardonable, as, from my knowledge of the judgment and ability of some of the gentlemen from whom they are said to emanate, I am thoroughly persuaded that one year's local observation would render it evident to themselves that, from the readiness with which the liver sympathises under a tropical sun with diseases originating in other organs, the *prudent* use of mercurial medicines becomes indispensable in the East and West Indies much more frequently than it can possibly be demanded in the more favourable climate of Great Britain.* Without, however, having had an opportunity of acquiring actual experience, or, perhaps, merely from a hurried visit of a few short months, to presume to disseminate censures on the practical methods of men who, educated in the same school with themselves, have subsequently passed a series of years in the laborious investigation and treatment of tropical disease, is most unwarrantably to arrogate *superiority* in matters where it is impossible that even *equality* should exist—to claim a right of dictation upon points of importance, regarding which, on entering this field of practice, they would probably find themselves at a loss to form a competent opinion even for their personal government, when called upon to prescribe." 453.

---

"* A leaf out of our book would not, however, be *on all occasions* without its use even to London physicians. For example, had an *experienced* tropical practitioner been in charge of the Milbank Penitentiary, he would at once have arrived at what Drs. Latham and Roget so tardily discovered, and thus have proved instrumental in saving many lives. I have repeatedly met with the particular species of purging which prevailed in that establishment, but chiefly in debilitated habits. An occasional dose of calomel, with blue pill and opium (in proportions varied according to circumstances), every third hour regularly, and alternated with the camphorated mixture, have been in my hands the successful remedies. It is not, perhaps, generally known, that camphor possesses a restraining power over the purgative and griping effect of calomel and blue pill, equal almost to that of opium itself; and I have seen the mistura camphoræ, with the addition of a few drops of tinctura opii to each dose, arrest protracted diarrhœa, which had resisted cretaceous mixtures, &c. in quantities almost as large as those issued by Mr. Pratt."

After protesting against the imprudent and indiscriminate use, or rather abuse of mercury, to which that medicine, in common with all others, is liable, our author returns to the more minute consideration of the method of treatment which he has found most effectual in hepatic ileus.

He first administers ten or fifteen grains of calomel alone, and then proceeds with five grains more, combined with an active cathartic, every third hour, interposing some purgative mixture, provided the stomach be sufficiently retentive. In recent attacks Dr. M. has sometimes succeeded at once, by a full dose of the submuriate, followed by an ounce or an ounce and a half of the oil of turpentine. But such success is not often to be looked for, and the plan above proposed must be steadily adhered to, varying the proportions of the calomel according to circumstances, and watching the accession of ptyalism, as absorption will go on for some time after the mercury is discontinued. "But the grand object of the treatment must never be lost sight of—which is, by means as gentle as may be devised, to bring the system *as speedily as it can be accomplished with safety*, under the decided influence of mercury, which would appear to communicate such a salutary impulse to the whole biliary apparatus, as instantly and completely pervades its obstructed channels and evacuates their viscid contents."

When the spasmodic affection of the intestines becomes severe, and when vascular excitement has been set up, immediate recourse should be had to the lancet, which often acts like a charm in removing the retching and abdominal pain. Mercury and the lancet may, in fact, be rendered mutually subservient to, and co-operative with, each other, in the process of cure.

Dr. M. makes many sensible remarks on certain adjuvants, as the warm bath, &c. but these need not detain us here.

We fully coincide with our ingenious author in his method of treatment—and we are much disposed to think that his theory of the disease is correct, not only as respects the dry belly-ache of the West Indies, but also the colica pictonum of the mother country. In the latter disease we have the strongest evidence of derangement of the hepatic *function*, among the very earliest phenomena—which derangement we firmly believe to be a cause, rather than a consequence, of many of the enteric symptoms which afterwards absorb so much of the practitioner's notice. We have paid attention to the disorders of painters, and we have observed that lead is as injurious to the functions of the liver, as it is to the nerves—and we have long ago concluded that the obstinate constipation and pain of the bowels were secondary or ternary links in the chain of morbid action, rather than primary. Finally, the treatment recommended by Dr. M. for hepatic ileus, is, in fact, the most certainly efficacious in the painter's colic of Europe—a strong proof of their having *one* pathological character in common at least.

---

2. *Diffuse Inflammation of the Cellular Tissue.** This dangerous

* Dr. Scott. Ed. Journal, October, 1825.

disease, as our readers know, has lately occupied the attention of many medical practitioners, who, as usual, are not agreed either as to its nature or treatment. Dr. Scott has recently published some cases of the disease, in our respected cotemporary of the North, which require no notice here; but his concluding or summary observations, respecting the treatment, are deserving of attention.

That which he has found most successful, when the disease arises simply from irritation, the individuals possessing tolerable strength, and the affection being as yet local, was early general bleeding, sometimes repeated, according to the violence of the symptoms, followed by leeches and cold applications, with purgatives. As soon as the affection reaches the arm-pit and adjacent parts, he considers all attempts at resolution as useless. Heat and moisture are then to be applied to produce or accelerate suppuration. When the affection has much tendency to spread, he employs blisters, and sometimes the caustic potash, with the view of exciting cuticular inflammation; and thereby limiting the diffusive character of the complaint by the effusion of coagulable lymph. Poultices are to be applied after these, and the parts to be incised as soon as possible, as the effusion of even serum is serviceable. Evacuations are to be procured by Epsom salts and tartar emetic from the beginning. When the irritability of the system is great, he gives laudanum and antimonial wine, allowing a cooling nutritious diet all the while. When suppuration is actually established, he gives porter or wine, with as much food of easy digestion as the patient chooses to take. In cases of dissection-wounds he believes there is a morbid poison introduced, which acts as a sedative on the whole system, and, consequently, he is more guarded in respect to sanguineous depletion, and inclined to adopt Mr. Shaw's recommendation of porter and beefsteaks. He advises, however, a middle course to be steered between depletion and stimulation.

In the above remarks we agree generally with the author. The fact is, that, in this complaint, no general rule can be laid down, and we must watch the symptoms as they arise, obviating them according to the state of the constitution and the progress of the affection—always bearing in mind, however, that inflammation of the cellular membrane, especially when arising from dissection-wounds, is not to be cured, like some other phlogoses, by decisive and reiterated depletions from the sanguiferous system. But, on this subject, we have said enough on former occasions.

---

3. *Traumatic Tetanus in the Horse.* Mr. Egan, of the 12th Royal Lancers, has published a successful case of this kind in the Lond. Med. Journal, for September last, which deserves a short notice. This disease appears to be of no very unfrequent occurrence in our cavalry during peace, although rare in civil life. It is equally fatal, Mr. E. observes, in the horse as in man. Our author and Mr. Castley, (the Veterinary Surgeon of the Regiment) have examined many horses who died of tetanus, and the latter has often found the theca of the medulla spinalis discoloured, as well as its covering derived from the pia mater, both exhibiting unequivocal marks of inflammation. Mr. E. can also testify that the liver, and, occasionally, the lungs, have been often found more or less diseased.

The horse, which was the patient in the present instance, had a wart torn off his abdomen while leaping a wall, and also received a contusion and slight laceration in his hind leg. This happened on the 10th April, 1824. In three days he was apparently well, and was afterwards exercised daily till the 28th, when his abdomen appeared very much tucked up, as if he had been some time without drink. His tail also began to quiver. 29th. The horse was worse. Masticated his food with difficulty, and his tail shook very much. Three quarts of blood were immediately abstracted. By 8 in the evening he exhibited unequivocal symptoms of tetanus and partial trismus. Venesection to 3 quarts; and a purgative was vainly attempted to be given by the mouth. The entire vertebral column, to the tail, was rubbed with the unguentum lyttæ. The throat, jaws, and the originally injured limb, were also blistered. Purgative enemata were administered. In the middle of the day the poor animal was seized with a paroxysm of acute tetanus and trismus, while a discharge of artillery was taking place in the neighbourhood. 30th. The vesicatories had acted well, and the tetanic symptoms were milder than yesterday. Cold affusion was tried, but aggravated the symptoms. May 1st. By desire of Mr. Castley, half an ounce of extract of opium was placed in the rectum as a suppository. The animal was turned out in the night, which was rather wet and cold. 2nd. Tetanic symptoms less acute, but more constant: trismus complete. No motion from the bowels for four days. 3rd. Tobacco infusion was now determined on. One ounce of leaf-tobacco was infused in a quart of boiling water, and, when cool, was thrown up per anum. The tetanic paroxysms appeared less acute, and a discharge of dark fluid fæces took place. The enema was repeated in the evening. The horse got down a quart of drink this day. 4th. Half a pound of tobacco was infused in two quarts of water and thrown up. The greater part of it was retained for ten minutes. It then produced a large fæcal discharge, and considerable nausea. The same tobacco was infused over again, and thrown up. The horse had only one tetanic paroxysm this day—trismus as before. He swallowed two quarts of water this day. 5th. No tetanic paroxysm— moves his jaw a little—and begins to pick at the hay, but cannot swallow it. 7th. There was a relapse, and exasperation of all the symptoms. More tobacco enemata were exhibited. These were repeated on the 8th, 9th, 10th, 11th, 12th, and on till the 15th, when the deglutition of fluids was perfectly restored, and no paroxysm of tetanus occurred. From this time the horse convalesced daily, and ultimately escaped from a disease which seldom spares his master.

This case comes in as corroborative of that published, some time since, by Dr. O'Beirne, of Dublin, and which our readers are already acquainted with.

4. *Cholera Morbus.** In our article on the cholera morbus of India, in this Number, we overlooked the little work of Dr. Ainslie, the title of which is below. Dr. A. does not appear to have had personal knowledge of any but the *sporadic* forms of the disease, but, as President of a Board at Madras, who were authorized to enquire into the late epidemic, he must have had access to all information on the subject. The jet of this interesting and erudite little volume may be stated in a small space. Dr. Ainslie avers that the first ejections from the stomach, in the cholera of India, if chemically examined, "will be found to be always more or less of an acescent nature." At this period there is no bile in the ejected matters. But, at length, "from the painful and frequent exertion in retching, bile, by regurgitation, finds its way into the stomach, and appears to give the first check to the disease by (allow me to say) somehow correcting, to a certain extent, the immediate cause of the sickness, which I believe to be an acid, and one so tenacious, that simple dilution has, nine times in ten, no effect whatever in dislodging it."

Our author believes, and in this we are disposed to agree with him, that epidemic cholera does not differ, in essential pathological character, from the sporadic disease, but only in *grade*, depending on various causes, many of which are, at present, beyond our cognizance. Our author's etiological creed may be gathered from the following short extract. "As far as I could judge from the data before me, it is not contagious. I at one time supposed that it might owe its origin to a peculiar distemperament in the atmosphere—a certain subtle something, not in chemical solution with the component parts of the air, for then there might be a chance of detecting it, but rather suspended in it and conveyed by it in a way that we are altogether unacquainted with." This theory (or rather confession of our ignorance) is, in our humble opinion, the nearest to the truth after all—but it has been lately relinquished by our ingenious author for another, but whether for a better, we will leave it for time to decide. After reviewing the various theories which have been broached by different writers, as to the *remote cause* of cholera morbus, Dr. Ainslie comes to the conclusion, though with proper diffidence, that "it is a somehow altered, diminished, or perverted distribution of the galvanic fluid, which particularly exerts its influence on this occasion—which further produces the sinking of the powers of animal life—in a word, the disease in question." In thus stating the naked fact—or rather opinion of our ingenious author, we acknowledge that we do him injustice in withholding his reasons for coming to this conclusion. But our narrow limits, and the great space which we have already dedicated to the subject of the Indian cholera, must plead our excuse. The small and unostentatious volume of our author is of easy access to every enquirer. We, therefore, hasten to the most important topic of all—the treatment recommended by Dr. Ainslie.

---

* Observations on the Cholera Morbus of India. By Whitelaw Ainslie, M.D. M.R.A.S. Octavo, pp. 170. 1825.

This may be readily anticipated from what we have stated in the beginning of this article. Having observed that the ejected matters were of an acescent nature, and that the aliment previously taken was generally an acid or acescent substance, such as limejuice, unripe fruit, crude vegetables, &c. Dr. Ainslie lost no time in having recourse to antacids, and generally gave preference to the subcarbonate of magnesia in a full dose—seldom less than two drachms and a half or three drachms, which he found a more certain remedy than the liquor potassæ or the subcarbonate of potash—in fact, says Dr. A. "so effectual was the remedy, that I found in very few instances indeed that I had occasion to repeat it." Dr. A remarks that the offending acid was by this means neutralized—the distressing vomiting ceased—the patient had, perhaps, a few loose stools—a reaction took place in the frame—the natural warmth was again restored to the extremities—the pulse became fuller and slower—and a tranquil sleep soon supervened to crown the whole, from which the patient never failed to awake free from complaint. By means of this simple antacid, our author "hesitates not to say that he has saved many hundred lives." Since his return to England he has ordered it with equal success—and he appeals to "one of the most distinguished physicians in London, whether or not he does not find the antacid just named an absolute *specific* in cholera morbus, provided it is administered in time."

On these statements we can have nothing to say. We have lived long enough in the world to be a little sceptical when a *specific* is proclaimed for any disease except syphilis and psora. We can have no hesitation, in the mean time, to say, that the remedy recommended by Dr. Ainslie is one which can have no other bad effect, at the worst, than the loss of time in exhibiting a better.

Dr. Ainslie is already favourably known to the world as a man of literary, scientific, and professional attainments. The little work which we have thus cursorily noticed does not detract from the previous reputation of the author.

---

5. *Epilepsy.* Dr. Williams, of Liverpool, has lately published a curious case of irregular epilepsy in a female of 11 years of age, which presented a host of anomalous symptoms, such as young females occasionally exhibit, confounding all systems of nosology, and puzzling the most experienced practitioners. First, Miss Bromhead had cramps in the upper and lower extremities—then lancinating pains in one temple and eye, with fixed acute pain in the epigastrium—next convulsive fits, recurring at intervals of an hour, with muscular agitations shifting from place to place in the body. These and several other abnormal symptoms disappeared for ten or eleven days, after a dose or two of oil of turpentine—but whether as a consequence or a mere sequence, we should not like to swear. Be this as it may, the fit returned, after the abovementioned interval, with redoubled violence, lasting two hours, and leaving the girl *speechless*, with "the full command of her tongue." The aphonic state soon disappeared, but left a host of morbid symptoms, more of the choreal than the epileptic character. After a series of ever-changing phenomena, among which were the loss of sight in one eye and the loss of hearing in one ear, she was put on a course of argentum nitratum, having been first affected constitutionally by mercury. In three days the fits ceased, and, in short, all the other alarming symptoms vanished. The nitrate was continued for some time, and the girl recovered. It will hardly be contended that three days of the argentum nitratum operated this magic cure. We have much doubt whether medicine had any hand in the fortunate change, having seen such wonders performed *by Nature* in girls of a tender age. For the honour of the healing art, however, "tell this not in Gath." Let us claim all that is due to physic—and also a little of what Dame Nature can easily spare on such occasions.—*Ed. Journal, October.*

---

6. *Chorea.* Dr. Venables has written a long paper, and published several cases, to prove (what we think few will deny) that chorea is not always an idiopathic disease—but often a secondary affection, dependent on some more general disorder of the system—especially chylopoietic derangement.

The cases published by Dr. V. evinced disorder of the digestive organs, and the choreal symptoms were removed by purgatives, mercury, bitters, bleeding, (local or general) and blisters, with proper attention to diet.

These cases go in confirmation of Dr. Hamilton's theory and practice, which have been long acted on—though the theory is often incorrect and the practice ineffectual.

---

7. *Diabetes—Venesection.* In one of the late Numbers of Magendie's Journal, M. Lefevre has related a case of diabetes, cured (as is said) by the means once extensively tried, but now nearly abandoned in this country—we mean venesection. The patient was a female, 50 years of age, of apparently good constitution, and who had laboured under diabetes for some time previously to application to Dr. L. She was in the habit of drinking from 12 to 15 pints of fluid in the day. Her sleep was interrupted, and, when she awoke, her tongue was always dry. Her skin was rigid and rough—no perspiration—constipation obstinate—appetite bad—sight weak—thirst incessant. The catamenia had ceased the preceding year. The urine, which exceeded the drink, was analyzed, and found to contain much sugar. Twelve ounces of blood were taken from the arm on 14th of June, 1824. There was no inflammatory buff, but the relief was immediate. She slept better—and found herself more active and comfortable, with diminution of thirst, moisture of tongue, &c. Animal food was prescribed, with equal parts of milk and lime water for drink. She took ten grains of ipecacuan (a pretty good dose !) at night—and calomel and colocynth pills to remove constipation. The hot bath was ordered to be used every evening. This not proving efficacious in bringing on perspiration, the vapour bath was used, and with success. The patient was bled a second time, to the same amount. From this time she rapidly recovered—the urine being diminished to the natural quantity.

It requires but a moment's glance at the history of the case, to be convinced that two bleedings, of twelve ounces each, could have had but a very trifling share in the removal of the complaint. The purgatives, the warm baths, and the animal food diet, were the remedial agents which, no doubt, effected the cure. In most of the cases of diabetes, which have fallen under our own notice, there was incurable disease of some internal organ, generally the lungs. Under such circumstances, we consider the diabetic affection as merely one of the means which nature takes to work the final overthrow of the fabric—though probably as a means of procrastinating the fate of the individual, at the same time.

---

8. *Croup.** Although the general property of serous membranes, when inflamed, is to throw out fibrin—and the mucous membranes pus; yet we see the peritoneum and pleuræ secrete a puriform fluid, in certain states of phlogosis, while we observe, in croup, the lining membrane of the trachea throw out a fibrinous crust. In slighter degrees of inflammation, however, it is the reverse, as we commonly see.

Mr. Mackenzie, the able and ingenious Andersonian Professsor of Anatomy and Surgery, in Glasgow, has drawn the attention of the profession to a point of practice, as well as a pathological observation, in croup, which are well deserving of notice.

"The fact to which I refer is, that the exudation of fibrin very frequently commences on the surface of the tonsils, thence spreads along the arches of the palate, coats the posterior surface of the *velum palati*, sometimes surrounds and incloses the uvula; and at last descending, covers the internal surface of the pharynx and œsophagus, the larynx and trachea. That this is the frequent progress of the fibrinous exudation, I am convinced, from the careful and repeated observation of the phenomena during life, and upon dissection.

"What is of much more importance, however, than the observation of a pathological fact, is the ascertained efficacy of a means of cure for this disease. Not merely have I repeatedly found the application of a solution of nitrate of silver completely successful in removing the fibrinous crust, covering the tonsils, velum and uvula, but I have been led to attribute the rapid alleviation and ultimate removal of all the other symptoms to this remedy, even in cases in which, from the severity and peculiar signs of the complaint, I had no doubt that fibrin had already exuded from the lining membrane of the larynx and trachea.

"The solution which I employ is a scruple of nitrate of silver in an ounce of distilled water. By means of a large camel hair pencil, this solution is to be freely applied, once or twice a day, according to the severity of the symptoms, to the whole lining membranes of the fauces. The surface of the tonsils, or wherever else the fibrinous crust is actually

---

* Mr. Mackenzie. Ed. Journal, April, 1825.

in view, will of course be particularly attended to; but I do not hesitate to push the pencil to the lower part of the pharynx. This remedy, so far from being productive of any irritation, beyond the mere mechanical and temporary one attending its employment, uniformly alleviates the symptoms of croup, such as the difficult respiration, the barking cough, and the peculiar anxiety of the little patient. It has evidently such an effect upon the diseased surfaces, both those which it actually touches, and those which are continuous, as to induce them to throw off the false membrane by which they are covered, and it appears also to prevent the farther progress of the exudation." 295.

We hope the experience of others will confirm the therapeutical observations of Mr. Mackenzie.

---

9. *Hydrocephalus cured by Pressure.* We are extremely sceptical as to the truth of those cures of hydrocephalus, which have been attributed to external pressure. We do not mean to impute any thing like wilful misrepresentation to the narrators—God forbid. But we may be permitted to think that *they* are not exempt from the errors, into which it is the lot of human nature too often to fall. We think they have been deceived in their estimates of the nature of the cases detailed—and this, we hope to be permitted to do, without giving any offence to individuals. But as our scepticism may be unfounded, in turn, we shall never fail to record that which militates against it, as well as that which tends to strengthen it.

Mr. Barnard, of Bath, has published the case of a child, 18 months old, who was brought to him, on account of the size of his head, which had increased much of late, especially during the preceding fortnight. The bones were much separated, "*and the fontanelles distended by the included fluid.*" Does Mr. B. mean to say, that the fluid could be distinguished? was it internal or external hydrocephalus? These questions cannot be determined by the paper. There were frequent convulsions—stomach and bowels much disordered—excretions unhealthy. For three or four days he tried the usual means of remedying the disordered secretions; but being unsuccessful, he directed his attention to the head. He ordered it to be closely shaved—and then he strapped it tightly with adhesive plaster. "In the course of *three days*, evidence of benefit was apparent to all." The convulsions ceased—the secretions were improved—the head diminished in size. From this time the child rapidly recovered—the adhesive straps being, from time to time, renewed—the cure was complete in a month.*

---

* The following passage from Dr. Baillie, whose authority is not of the meanest, may be properly introduced here.

"After water has begun to accumulate in the head of a child, before the bones of the cranium are closely united, it is well known that the effusion may ultimately become very large, that the head may be proportionally increased in size, and that life may be continued under such circumstances for many months, or even years. When the bones of the scull have once

We leave the reader to draw his own conclusions in this case. Our own are already made known. *Med. Repos. Sept.* 1825.

10. *Croton Oil.* In the August No. of the Medical and Physical Journal is a report, from Ed. Tegart, Esq. Inspector of Army Hospitals, on the oil of croton, as employed by the military practitioners and others in the West Indies. By this report, the medicine appears to have been highly beneficial, not only in acute affections, requiring depletion, but as a stimulant in chronic visceral complaints. Where water had been effused in the chest or abdomen, it proved a powerful hydragogue. At first, the reporter only used the oil as a purgative, but experience in the West Indies soon convinced him that it was an excellent febrifuge, after the first object had been attained. "Dissolving it in the spirits of wine, and then diluting it in any palatable vehicle, so as to give half a drop for a dose, every three or four hours, it keeps the bowels open, increases the urinary discharge, and relaxes the skin:—these are important objects to gain at the commencement of all febrile affections." The reporter has found this medicine extremely powerful in the yellow fever of the West Indies. There being generally much gastric irritability, with obstinate constipation of the bowels, he found it, he says, an important remedy for the removal of both. Considering the acrid as well as drastic quality of the croton, this experience of Mr. Tegart does not say much for the gastro-enteritic pathology of the disease, as maintained by the French school. But we fear our author has over-rated the powers of this new remedy—a circumstance which almost always takes place, when a new article is introduced in the materia medica. We think the said croton oil is a very useful adjuvant to other purgatives, by heightening their powers, without producing the disagreeable effects resulting from its solitary employment. This is the result which we believe it will ultimately retain—if at all retained in the list of medicinal agents.

11. *Hydrophobia.* Two cases of this dreadful disease, are related in one of the late Numbers of the *Archives Generales.* In the first case, death took place, though in a more protracted manner than usual. There was nothing particular in the symptoms during life, and therefore, we shall only state some particulars of the dissection. The membrane of the palate was inflamed, and lined with a kind of false membrane.

become closely united, if Hydrocephalus take place, it almost constantly happens, that the bones still remain firmly joined together, that the quantity of water in the ventricles is comparatively very small with what it is in those cases where the bones have never been united, *that the head undergoes no increase of size, and that life is very soon destroyed.*"—*Wardrop's Ed. vol.* i. *p.* 12.

Thus we see that the very circumstance of the sutures opening, and the bones giving way, prolongs life, and *vice versâ.* What then is to be expected, when pressure *ab externo* is added to pressure *ab interno* on the substance of the brain?—*Rev.*

The pharynx and tonsils were red, as were the œsophagus and mucous membrane of the stomach. Marks of inflammation were also found in several parts of the intestines. The lining membrane of the trachea and all its ramifications was red—the lungs gorged with blood, as were the vessels of the brain. The spinal marrow, throughout its whole extent, as well as the nerves that issue from it, were perfectly natural.

In the second case, related by M. Cassan, a boy, 14 years of age, was bitten by a dog, whose state was unknown, on the 14th June, 1824. The wound was cauterised with red hot iron, and a blister afterwards kept on the part. Nothing occurred to disturb the boy's health till the beginning of August, when he became irritable, melancholy, and taciturn. On the 7th of August, hydrophobia was declared, and next day it was unequivocal. It was now determined to try the effects of vinegar in this hitherto fatal disease. At first the boy consented to wet the corner of his handkerchief in vinegar, and wipe his lips therewith. He then tried to swallow some bread sopped in the same, but soon threw it out of his mouth with horror. Soon after this he was seized with ptyalism of a viscid and frothy kind. Vinegar mixed with water was then administered by injection, to the amount of a pint and a half of the former, in the space of three or four hours. The first injection was returned, with fœcal matters. A part of the second, and also of the third was retained. The pulse fell in frequency, but became very hard. The other symptoms continued unaltered. He tried to swallow some drops of water, but he said it burnt him. Gradually, however, the power of deglutition improved. More vinegar was exhibited by the mouth.* The nervous symptoms diminished, but vomiting came on, and became incessant. He died at midnight. Dissection did not present any thing that is worth commemorating.

M. Cassan and his father, who attended the unfortunate boy, were decidedly of opinion that the vinegar greatly ameliorated the hydrophobic symptoms, and that it would have saved his life, had it not been injudiciously administered by the anxious parents of the patient. It need hardly be remarked that vinegar was long ago held forth as a specific against hydrophobia. But we greatly fear, that as the nature of variolous inoculation is to produce pustules, so is that of the hydrophobic poison to produce death. When either of the poisons is once introduced into the system, we have no power to stop its progress!

---

12. *Stomach Pump.* About twelve months ago, Mr. James Bryce, of Edinburgh, announced an apparatus for throwing liquids into, and extracting them from the stomach, which is certainly much more simple than the stomach pumps of Weiss and Reid. As to the point of its being equally effectual, we cannot speak. All we have to do, at present, is to make the thing known. The apparatus is neither more nor less

* This was done by the parents, in the absence of the medical men. They ignorantly exhibited it undiluted, and brought on the vomiting, with perhaps, inflammation of the stomach.—*Ed.*

than a bladder affixed to one end of a tube, the other end of which is introduced into the stomach. When the bladder is filled with any fluid, and raised above the patient's head, the fluid runs down into the stomach—and when the same bladder is lowered, so as to be under the level of the stomach, the fluid runs back again, on the principle of a syphon. For a more minute account, we refer to the Edinburgh Journal for January and April 1825.

Since the publication alluded to, Mr. Bryce informs us that he has made some improvements on the construction of his instrument. It consists, at present, of an œsophagus tube, about 24 inches long, but, instead of the tin tubes formerly recommended, he has substituted a flexible air-tight tube, about three feet in length. This tube is made of coarser materials than the œsophagus tube, and resembles those used by tobacconists. It is mounted at one end with a small brass tube fitted to a similar mounting on the extremity of the œsophagus tube, so as to slip in and out at pleasure, and when joined to be air-tight—and the other end of this tube is fixed to a bladder, of size sufficient to hold about a quart of fluid, having a ring and stopper at its extremity, for the purpose of filling or of emptyiug it at pleasure. It must be attended to, that the calibre of this flexible tube, and of the brass mountings, should be somewhat larger than that of the œsophagus tube. Œsophagus tubes, of various sizes, may be necessary for patients of various ages; but they may all be fitted to the same brass mounting of the flexible tube. It is evident, that by fitting an ivory pipe to the flexible tube, any quantity of fluid can be thrown into the rectum and bowels, and again extracted from thence at pleasure, by the syphonic power above alluded to.

The instrument is constructed by Mr. M'Leod, of Edinburgh, and by Mr. Evans, of London, where it may be seen. For more details respecting its application, we refer to the two numbers of the Edinburgh Journal quoted above.

---

13. *Hydrophobia Prevented.* If we may credit certain statements in some German journals, we have a sure preventive of the most frightful and hitherto fatal disease to which humanity is liable. It is said that both in Zurich and Breslau a nearly similar plan has been long adopted, and with entire success. The Zurich plan is the oldest, having been employed since the year 1783—the Breslau bears date of 1797.

1. *Zurich Treatment.* Deep scarifications of the bite, besmearing it with the pulvis lyttæ—perpetual blister to the neighbouring parts, keeping both open for the space of six weeks. In the mean time, mercurial frictions are to be used till salivation approaches. The adult is to take internally, for three weeks in succession, every second morning, five grains of powdered belladonna—or, as a substitute, if the patient comes under treatment a few days after the accident, calomel should be administered. When the belladonna is given, the patient should experience, from each dose, the symptoms of incipient intoxication, after which he should take a diaphoretic, with copious draughts of tea or other diluent. He should keep in bed for the first month, and abstain from animal food.

According to this mode, 233 persons, bitten by rabid animals, have been treated in 42 years. Many were admitted into hospital, from the second to the fourteenth day after the accident—and some so late as the eighth week. Of this number only four died—two on the second day after their admission, and consequently after the disease had been established. The other two were bitten in parts where scarifications and the means prescribed could not be employed—being in the eye and in the mouth.

2. *Treatment of Wendt.* Wendt recommends the wound to be filled with powdered cantharides, and a copious discharge to be kept up for six weeks. Also a smart mercurial ptyalism to be maintained during that period, by means of frictions externally, and calomel administered internally. Of 106 persons bitten by mad animals, and thus treated, between the years 1810 and 1823, two only died. Seventy-eight others were treated in this manner, but there was reason to believe that the animals by which they were bitten were not rabid.

After making ample allowance for many cases, where the anima might have only been *supposed* to be mad, yet it is highly probable the the prophylactic treatment pursued in the above instances, was often the means of warding off the terrible consequences, which generally result when no preventive measures are taken.

We may remark, however, that where free *scarifications* are practicable, *excision* of the bitten part might be performed, which would be a surer method. The discharge could be maintained equally well after excision as after scarification. As to the internal treatment, it is evident that mercurialization is the main point—and this measure has been found successful, on a large scale, in the East Indies, more than 30 years ago, as published by Mr. Daniel Johnson, of the Bengal Medical Establishment.* This Continental corroboration of Mr. Johnson's prophylactic method will, we hope, induce our brethren to give a trial to the plan found so efficacious elsewhere.

---

14. *Sanguineous Apoplexy.* Mr. Abraham, of Worcester, has related one of the most wonderful cases of apoplexy, which we have ever seen or ever read of, in the October number of the Edinburgh Journal. A man was seized "with a fit, apparently of syncope," which gradually *increased*, till his limbs "became *rigidly* extended." In this fit of syncope, Mr. A. found the patient "breathing slow, his eyes shut, his skin

* For a full account of Mr. Johnson's Paper on Hydrophobia, see the 3d. Edition of Dr. James Johnson's Influence of Tropical Climates on European Constitutions.

moderately warm, his pulse fluttering, &c." In this same fit of syncope or "sanguineous apoplexy," in which he seemed "entirely senseless," our author called to him in a loud voice, to say whether or not he understood the purport of his questions? The patient whispered, in a low tone, that he knew very well what was going on about him. He was asked to open his eyes—and open them he did. He complained of rigidity and pain in the region of the masseter, and oppression at the præcordia—in short, he gave such answers to Mr. A. as assured the latter, that his intellects were unimpaired. "The whole scene," says the surgeon, "was singular." In this desperate fit of "sanguineous apoplexy," our author abstracted about a quart of blood, when the patient candidly, but rather uncourteously, told his medical adviser "that he was worse." The surgeon then closed the orifice. Three or more colocynth pills were administered, with a sufficient quantity of calomel, and the patient soon recovered.

We should like to ask our respected cotemporary, what is the age of his correspondent, Mr. Robert Abraham, surgeon, and Member of the Physical Society of Guy's Hospital? We take him to be very young, otherwise he would have perceived that his patient's malady was seated in the stomach and not in the brain. He had dined on beef-steaks, with, no doubt, a good proportion of onions—he plainly told his surgeon that he felt "sea-sick and groggy," and the surgeon himself observed that "his eyes and countenance resembled those of a person intoxicated." When Mr. A. has lived 20 or 30 years more, he will have seen many cases of this kind, arising from food disagreeing with the stomach—and he will not consider a man in a state of sanguineous apoplexy, while he answers questions distinctly, with "intellectual faculties unimpaired." Neither will he set down a man as in a state of *syncope* with "his face turgid and suffused," and the heart and lungs in action —even if "his limbs became rigidly extended." What would the Society at Guy's have said to this case, in one of their Saturday-night's debates?

---

15. *Traumatic Tetanus successfully treated.* A case of this kind is an oasis in the desert of tetanic therapeutics, and the eager enquiry will be—what were the means used? Purgation by drastics, including croton oil, calomel, scammony, &c. in large doses, with mercurial ptyalism. The case was treated by Mr. Manifold and Dr. Briggs, of Liverpool, and published in the October number of our *senior* cotemporary of the Intellectual City—for we have now a *junior* also in the Modern Athens.

A youth of 15 years, had a compound fracture of the right thumb, which was going on well till after a long walk of 18 miles, when he became feverish, and was seized with tetanus the same night. He was conveyed back to the Liverpool Infirmary, and came under the care of Mr. Manifold. This gentleman exhibited smart doses of calomel and scammony, and ordered the thumb to be poulticed. It will be unnecessary to enter into the diurnal details in this place; we shall only state the results, and advert to the principal means. Thus, in one day, he took hydrarg. submur. et scam. ā ℈ij. gambogiæ ℈j. with four doses of the black draught. In addition to which, he took a tobacco injection, and a blister along the spine. These medicines were continued day after day—often with no effect, and seldom with any thing like a full purgation. On the fourth and fifth days of the treatment, ptyalism came on. Croton oil had also been administered. There were now more abundant fœcal discharges, and the tetanic symptoms were considerably mitigated. The purgatory plan was strenuously pursued, with ultimate though tardy success. The treatment was commenced on the 17th April, and on the first of May he was able to walk to the market-place and back without spasms or much fatigue.

We need hardly say that this practice is not new, as it has been often put in force between the tropics, as may be seen in the writings of Drs. Dickson, M'Arthur, and some other naval and army practitioners. It has rarely, if ever, been so energetically put into execution in this country, chiefly, we believe, from the timidity, or, rather the prejudice, of European practitioners against large doses of medicines in desperate diseases. They shrink from the imaginary danger of a larger dose of medicine than they are accustomed to administer, but look on, with great boldness, at the approach of death from the disease! This last is a thing they are so *much accustomed to*, that it becomes a matter of course—and both the medical attendants and friends are consoled with the reflection, that every thing was done which skill and science could suggest—and so the remainder of the work is left to the undertaker. We know not when the scholastic prejudices about the operation of medicinal agents will give way to the light of truth. We, this very night (29th of October) heard it gravely asserted, in a medical society, by a man of science—that is an experimenter—that we should be very cautious how we increase the doses of medicines, since, by *doubling* the dose, we *quadruple* the common effects. This was asserted on the basis of *facts*—and all we have to answer is, that these facts are falsehoods.—They are falsehoods, because the effects of medicines on animals in *health* are no criteria of their effects in diseases. A man is seized with violent spasms in the stomach or bowels, and he takes a drachm of laudanum and two or three glasses of hot brandy and water before these spasms are allayed. He rises in the morning without a head-ache, or any perceptible effect resulting from what, if taken in health, would have made him ill for two days. All these results of observation at the bed-side of sickness, go for nothing with the experimenter, who confidently and proudly erects his *careful deductions* on the veriest basis of error. We shall revert to this subject again and again, and we are not without hopes of counteracting, in a considerable degree, such fallacies and obliquities of the human intellect.

---

16. *Erysipelas.* In the October Number of the Ed. Journal, there are two communications on the treatment of erysipelas—one from Mr. Maclean, of Kilmalcom—the other from Dr. Burrell, Assistant Surgeon of the 72nd Regiment, at Edinburgh. Mr. M. gives the detail of one case, where general blood-letting and active purgation, with cold applications, conducted to a successful issue an erysipelas of the face. Dr. Burrell details two cases, where extensive leeching, the warm bath, and fomentations, led also to recovery. In the latter cases, however, the erysipelas was a supervention on other diseases, and, therefore, not of an idiopathic nature. The treatment pursued by these two gentlemen cannot be said to be identical, though, in the hands of both, successful. We all know that many practitioners pursue a diametrically opposite methodus medendi, and they, too, cure their patients. Thus, at St. George's Hospital, where erysipelas may be said to be at least as regular in its visitations as the medical officers themselves, the cordial plan of treatment is generally adopted, and the majority of the patients recover. But, says Dr. Burrell, in his comment on the text of Dr. Good, erysipelas is an inflammation—" and if not made to yield to an antiphlogistic plan, *cannot reasonably be expected to give way to any other*." This erysipelas must, therefore, be an *unreasonable* disease, and, in despite of theory, does give way very often to other than depletory plans of practice.—Mr. Maclean skilfully shields his bold measures in erysipelas under the sanction of the Editor to whose journal he addresses his paper. " In conclusion," says he, " I *cordially* agree with Dr. Duncan in thinking that—' there is nothing in the nature of erysipelas essentially different from other inflammations'—and, in my opinion, we ought to treat it as fearlessly as we do other affections belonging to that class of diseases." With submission to Dr. Duncan and Mr. Maclean, we would take the liberty of doubting the identity of erysipelatous and phlegmonous inflammation; for example, the *former* is very generally a constitutional, the *latter* a local disease. The one has a constant tendency to spread—the other to circumscribe its sphere of operation. The erysipelas is accompanied by a typhoid, the phlegmon by an inflammatory pyrexia—the *former* often ends by transference to internal structures—the *latter* hardly ever does so. Now, if we are to break down distinctions so marked and unequivocal, and amalgamate the diseases and their modes of cure, why make any distinctions at all, but treat all diseases alike? Why has Dr. Duncan given a valuable paper on " Diffuse Inflammation of the Cellular Membrane," and suggested a modified treatment in the same, if he identifies erysipelas and phlegmon?

We make these comments, not with the view of preventing due depletion in certain cases of erysipelas, but to prevent the young practitioner from regarding this dangerous inflammation as essentially the same with common phlegmonous inflammation, and to be cured by simple energetic depletion. If he pursue the latter mode of practice indiscriminately, he will often have to deplore the loss of his patient's life, and perchance, in the end, of his own reputation.

---

17. *Sympathetic Amaurosis.** Mr. Wishart has lately recorded a

* Mr. Wishart. Ed. Journal, July, 1825.

very interesting case of this kind in the person of a young lad of nine years, who was brought to him from the country, completely blind of the *left eye.* No difference could be perceived between this and the other eye on the most careful examination. He had occasional headaches over that eye—and his digestive organs were evidently deranged, and the fingers of the *left hand* were often spasmodically drawn into the palm. The *left foot* was also similarly affected, the toes being turned in under the sole of the foot. The loss of sight was stated to have come on suddenly, about four months previously, on hearing of his grandmother's death. The nursery maid had been in the habit of giving him whiskey at night, to make him sleep—being herself fond of a drop of Ferintosh. Emetics—cathartics—calomel—a blister to the mastoid process. One morning, on awaking, he was found to be labouring under a smart febrile attack, and soon afterwards had a copious discharge from the bowels, containing numerous lumps of indurated fæces. By noon of that day "his sight was perfectly restored." The vision continued uninterrupted; but the blister was directed to be kept open for some time, and, as he had been much weakened by purgatives, bitter tonics were ordered.

This case requires no comment. It bears on another subject recently discussed—the carpo-pedal spasm of children, which we have long thought to be more dependent on gastro-enteric irritation than on *idiopathic* affection of the head.

---

### *Toxicology.*

1. *Poisoning by Laudanum.* Drs. Ollivier and Marye, of Angiers, have published a case of this kind in the Archives Generales, (April, 1825,) on which we shall make a few comments anon.

A young gentleman, 28 years of age, of robust constitution, and much addicted to gambling, swallowed an ounce and a half of laudanum, as exactly ascertained afterwards. This was at 8 o'clock in the morning of April 4th. He was not discovered till one o'clock, or five hours after the act was committed, when the following were the phenomena presented. Decubitus dorsalis—profound stupor, from which he could scarcely be roused by the loudest noise—face and lips pale—expression of countenance tranquil—*pupils excessively contracted.* When roused, the patient looked distractedly at the surrounding spectators, and observed that they appeared as if enveloped in a mist. His answers to questions were slow, but rational—the intellectual faculties not otherwise deranged—pulse hard, and 109—breathing free—no pain at the epigastrium—no nausea or vomiting—no stool, no urine—occasional tremor of the whole body; but no convulsion or loss of sensibility. The patient revived occasionally from his lethargic condition, and then fell back again into the same state. *Three grains of tartar-emetic were administered, in half a glass of warm water, followed by two other cups of warm water.* The patient took the cup in his hand, and drank with facility. At half past 3, P.M. ($7\frac{1}{2}$ hours after the act) there was no vomiting. (Who could have expected any?) A purgative lavement, which had been thrown up, was returned. The patient got up, with assistance, to the commode. He then fell into a profound stupor; yet, when roused, he answered distinctly. *The pupils were contracted to a point*—pulse 90 in the minute—respiration prolonged. Twelve ounces of blood were drawn from the arm, which was very florid, and soon coagulated. The patient obstinately refused to drink some coffee which was offered to him. At 5 o'clock (9 hours) the same state, except that the stupor was more profound. The pupils exceedingly contracted. When roused, the patient could still return distinct answers to questions. M. Orfila now saw the patient, and advised purgative lavements, with the administration of coffee and lemonade. He drank much of these. At 8 o'clock (twelve hours from the accident) the stupor still continued profound—respiration slow—only four or five in the minute—pulse 88—skin cold and dry—pupils still contracted. The patient could scarcely distinguish the bye-standers, though he knew them by their voices. The lavements had procured copious evacuations. Continues to drink lemonade and coffee. Ætherial mixture. At 15 hours after the accident, the breathing was slow and suspirious—the ideas incoherent—but he was still capable of taking lemonade and coffee. In the night he was delirious. After the expiration of 24 hours, the symptoms were mitigated. He was bled to 24 ounces, the blood being as florid as before. The patient entirely recovered, and no symptoms of a narcotic character remained after the end of 48 hours from the swallowing of the opium. The pupils all this time remained contracted, but afterwards regained their natural state of dilatation.

*Remarks.* The authors take great credit to themselves in the recovery of this patient after taking such a large dose of laudanum; but we are very far from joining in this self-commendation. The exhibition of three grains of tartar emetic, with the view of expelling twelve drachms of laudanum from the stomach, five hours after its exhibition, was a bold stroke certainly, and in this measure they seem to have expended their valour, for they made no farther efforts to dislodge the enemy! In two hours and a half more, and without any preceding evacuation from the stomach, they bled the patient—a measure calculated to promote the absorption of the poison into the system! At the end of nine hours, and still without any vomiting having been excited, M. Orfila ordered vegetable acids to be given—a measure calculated to heighten the deleterious effects of the laudanum, which was still in the primæ viæ, or circulating with the fluids of the body! It is true the patient recovered—but, in our humble opinion, it was, to use an antiquated phrase, "more by good luck than good guiding." We think a more mismanaged case could not well be placed on record, notwithstanding the following flattering opinion given of their own conduct. "On peut donc attribuer avec quelque fondement au traitement que nous avons mis en usage, la disparition rapide des accidens qui s'etaient manifestés."

M. Barreuil analysed the blood which was drawn from this patient, and appears to think that he has proved the existence of morphine in this fluid. It is probable that he is correct in this conclusion.

The obstinate contraction of the pupils in this case, is rather an interesting phenomenon, in a medico-legal point of view—the general opinion being that *dilatation* is a characteristic of poisoning by opium.

---

2. *Poisoning by Cantharides.* M. Julia Fontanelle has published a case of this kind, in a late number of the Revue Medicale, which we shall condense in this place. It is well known, that idiosyncrasy of constitution greatly modifies the effects of poisons, as well as of their more gentle neighbours—medicines. Thus, Amoreux relates the case of a delicate lady, who swallowed three drachms of powdered cantharides, while one of her friends took, by way of encouraging her, a very small quantity of the same material. Contrary to expectation, the *former* suffered only some ardor urinæ, and heat about the throat, which were dissipated by mucilaginous drinks and camphor; while the *latter*, who had taken so much less of the medicine, was reduced to the door of death, and narrowly escaped with life. We shall now proceed to M. Fontanelle's case.

*Case.* Banot, a young man, about 20 years of age, of strong constitution, was attending on his master, who laboured under low fever, and was taking bark in wine every two hours. Banot, in mixing up the fourth dose, put, by mistake, a packet of powdered cantharides (half an ounce) into the glass of wine, instead of a paper of cinchona. His master, being disgusted with the three doses already taken of the bark and wine, obstinately refused to take any more, and the servant, by way of encouragement, quaffed off the potion himself. In a few minutes, he felt a severe ardor urinæ, burning pain in the throat, and pain in the head. To these symptoms succeeded vomiting. The cause of the accident being soon discovered, he was conveyed to the hospital, and placed under the care of Drs. Martin and Caffort, who immediately administered six ounces of sweet oil, and put him into a warm bath. By these means, the symptoms were a little relieved, but the vomiting continued. Milk and mucilaginous fluids were given for drink and by way of lavement, while pills of camphor and nitre were prescribed. In the evening, or eight hours after the accident, there was bloody urine, with great ardor urinæ. Next morning, there was less discharge of blood, but the ardor urinæ, with strong priapism, continued. The same treatment was employed as before. *Third day.* The vomiting was much abated, and all the other symptoms diminished in intensity. It was 12 or 14 days before the patient was able to leave the hospital, which he did, quite cured.

It is doubtful whether the oil was useful or injurious. It is certain that it is a powerful solvent of the acrid principle of the cantharides.—*Rev. Med. September* 1825.

